# Evaluating the Role of Maximal Expiratory Flow at 25% (MEF-25) in Detecting and Managing Patients With Small Airway Disease in the United Arab Emirates

**DOI:** 10.7759/cureus.74319

**Published:** 2024-11-23

**Authors:** Aadil Ashraf Ahmed Shaikh, Mary Ann Boniface, Nida Naeem, Syed Ammar Husain, Leon Gerard D Cruz, Syed Arshad Husain

**Affiliations:** 1 Department of Pulmonary Medicine, King's College Hospital, Dubai, ARE; 2 Department of Medicine, University Hospitals Sussex NHS Foundation Trust, Sussex, GBR; 3 Department of Research and Innovation, Portsmouth Hospitals University NHS Trust, Portsmouth, GBR

**Keywords:** asthma, beclomethasone-formoterol, cough, inhaled corticosteroid, long acting beta agonists, mef-25, shortness of breath, small airway disease, spirometry, wheeze

## Abstract

Middle Eastern countries, such as the United Arab Emirates and Oman, are affected by frequent dust storms and extreme hot climatic conditions, which can exacerbate respiratory conditions. These environmental factors are particularly injurious to asthmatic patients, as they can aggravate small airway disease (SAD), leading to increased morbidity and healthcare challenges.

The evaluation of maximal mid-expiratory flow (MEF-25) as a diagnostic and therapeutic tool for early-stage small airway dysfunction is of significant clinical importance, particularly in hot and arid metropolitan environments where dusty conditions exacerbate pulmonary issues. This study assesses the value of MEF-25 in clinical practice for detecting SAD and investigates the effectiveness of inhaled bronchodilator therapy with extra-fine particles augmented by a spacer in helping to manage the symptoms of these patients.

This retrospective, single-center study was conducted at King's College Hospital Dubai, including 225 patients aged 18 and above, who presented with MEF-25 values less than 80%. Data were collected from the Electronic Medical Record system, including demographics, spirometry findings, and treatment outcomes. MEF-25 values were categorized into moderate (51-80%), severe (35-50%), and very severe (1-34%) grades. Statistical analysis was performed using IBM SPSS software.

Results revealed significant correlations between MEF-25 severity and various clinical parameters. Notably, Immunoglobulin E (IgE) and Fractional Exhaled Nitric Oxide (FeNO) levels showed an inverse relationship with decreasing MEF-25 values. Symptoms such as wheezing were more prevalent as MEF-25 values decreased, indicating that MEF-25 is a sensitive indicator of small airway dysfunction (SAD). Additionally, treatment with an inhaled corticosteroid (ICS) combined with a long-acting beta-agonist (LABA), using an inhaler with a fine particle size and augmented by a spacer device, demonstrated substantial and prompt improvement of symptoms. Follow-up data showed a high rate of symptom resolution within one to six weeks after treatment initiation.

This study underscores the importance of MEF-25 in the early detection of SAD in the setting of dusty, humid, and hot climatic conditions, and supports the use of beclomethasone-formoterol with a spacer as an effective treatment strategy in optimizing clinical outcomes. The findings advocate for the integration of MEF-25 in routine spirometry evaluation to enhance the diagnosis and management of SAD, particularly in environments prone to high amounts of respiratory allergens.

## Introduction

Small airways, defined as having diameters of less than 2 mm, are particularly vulnerable to pathological changes and occlusion, which can lead to respiratory complications [1]. The problem is intensified in locations where dusty and humid environmental conditions have a detrimental impact on the small airways, resulting in symptoms such as wheezing, shortness of breath, cough, and chest tightness.

The pathogenesis of small airway disease (SAD) involves increased mucus production, smooth muscle hypertrophy, and the presence of inflammatory infiltrates within the bronchial walls, which lead to a reduction in luminal diameter and an obstruction to the flow of air [2]. This condition significantly reduces the patient's quality of life and imposes considerable limitations on their daily activities. Therefore, it is crucial to promptly diagnose and effectively treat this condition.

Obstructive airway diseases, such as asthma, are typically diagnosed through clinical history, signs and symptoms, and lung function data derived from spirometry. Compared to impulse oscillometry, spirometry is more cost-effective, accessible, and provides easier interpretation of results [3], making it the preferred method among healthcare professionals.

Spirometry assesses lung volume change rates during forced breathing, beginning with full inhalation followed by extended forced exhalation until a plateau is reached, which are graphically recorded. Key measurements for obstructive airway disorders include forced expiratory volume in one second (FEV1), forced vital capacity (FVC), and the ratio of FEV1/FVC, along with maximal mid-expiratory flow (MEF, also known as forced expiratory flow, FEF) values at 75%, 50%, and 25% of exhalation. The value of MEF, which is obtained from the flow-volume curve, is expressed in liters per second. An isolated reduction in MEF25 indicates small airway obstruction, as the last 25% of vital capacity originates from the most distal airways (bronchioles), making it one of the most beneficial investigations for diagnosing pulmonary conditions with SAD.

Once the diagnosis of SAD has been established, the subsequent course of action for clinicians is to relieve the patient's symptoms by means of medication, preferably an inhaler in this case. The most effective inhalers are those with small particle sizes, capable of reaching and penetrating deep into the small airways. Many inhalation devices primarily deposit medication in the central airways, limiting efficacy in the peripheral airways. Reducing the mean particle size of inhaled drugs to 1.5-3 micrometers enhances drug delivery to these peripheral areas [4,5].

The preferred treatment for optimal control of symptoms associated with SAD involves the use of both a long-acting beta2 agonist (LABA) and an inhaled corticosteroid (ICS). The inhaler frequently employed for this purpose, and the one utilized in our study, is beclomethasone-formoterol. The extra-fine formulation of beclomethasone and formoterol in a pressurized metered dose inhaler has demonstrated effectiveness and tolerability, surpassing other fixed ICS/LABA combinations [6,7,8,9]. Beclomethasone dipropionate, a glucocorticoid, provides lung-specific anti-inflammatory effects, whereas formoterol, a beta 2 adrenergic agent, induces rapid (1-3 minutes) and sustained (up to 12 hours) bronchodilation. Combining these two ingredients in one inhaler efficiently delivers medication to the distal airways, significantly improving symptoms. Its effect is further amplified by the addition of a spacer device. A spacer is an attachment that extends and disperses the aerosol spray from an inhaler into a fine mist, facilitating slower and more even delivery to the lungs. This minimizes particle accumulation in the mouth, tongue, or oropharynx, reducing local side effects from medications and enhancing the concentration of medication inhaled directly into the lungs [10].

The objective of our research is to evaluate the function of MEF-25 in detecting and treating early-stage small airway dysfunction in clinical practice in the United Arab Emirates. Additionally, we aim to establish the effectiveness of combining beclomethasone-formoterol with the use of a spacer in managing this condition.

## Materials and methods

Site description****

Patients were selected from the Pulmonology Outpatient Clinic at King's College Hospital Dubai, a prominent private healthcare provider in the United Arab Emirates.

Procedure for ethical approval

Ethical approval was obtained from a local Research and Education Committee (approval no. KCH/MOI/740, May 2024) within the site of investigation in accordance with the ethical and legal guidelines in the United Arab Emirates.

Study design and study population****

This is a retrospective, single-center study aiming to investigate diagnostic and therapeutic considerations for small airway dysfunction in the population of the United Arab Emirates.

We reviewed patient charts from October 2023 to April 2024 using the Electronic Medical Record (EMR) system. Patients were screened using predetermined inclusion and exclusion criteria. Symptomatic patients aged 18 and above with MEF values less than 80% were included. Children and patients with a formal diagnosis of interstitial lung disease, COPD, or evidence of pneumonia were excluded. A total of 225 patients from various ethnic backgrounds from Dubai and neighboring emirates were ultimately included.

Data collection****

The EMR system (Cerner) was used to collect data including age, sex, ethnicity, pets, smoking exposure, presenting symptoms (cough, wheeze, chest tightness, shortness of breath), spirometry findings (FEV1, FVC, MEF25), FENO, IgE, and eosinophil counts. Information on previous asthma control, if available, and past prescriptions of inhalers and medications were also obtained.

A careful review of follow-up outcomes in symptoms (cough, wheeze, chest tightness, shortness of breath) was recorded from the physician’s notes after a minimum of one week. Additional information, such as peak flow meter readings, was included when available. Data were tabulated in password-protected Excel sheets while maintaining the anonymity of subjects.

Grading severity of MEF-25 dysfunction****

We graded the MEF-25 values of study participants into moderate (51-80), severe (35-50), and very severe (1-34) based on the informed consensus of the research team in accordance with existing evidence. The grading we adopted roughly follows the 'Interpretation of any spirometry findings based on FEV1' as published by Pellegrino R et al., in 'Interpretative strategies for lung function tests' [11].

Statistical analysis****

Our statistician analyzed this data using IBM SPSS software. Correlations across the different severities of MEF-25 dysfunction and variables such as sex, symptoms, smoking status, and pets were examined using the Independent Samples Kruskal-Wallis Test and Pearson's chi-square test. Further analysis was conducted using Phi and Cramer’s V statistical tests.

The frequency and prevalence of presenting symptoms and response to treatment were recorded and examined in this study population to obtain valuable information on treatment outcomes.

The mean, median, and mode of BMI, IgE, eosinophils, FENO, FVC, and FEV1 across the different severities of MEF-25 dysfunction were calculated to examine common patterns, if any.

## Results

Our results consisted of general patient characteristics, an analysis of qualitative variables across the three grades of MEF-25 severity, and an examination of the symptom-wise response of small airway disease patients to beclomethasone-formoterol.

General patient characteristics 

The general patient characteristics of our patient population are shown in Table 1. The mean age of our patients was 41.8 +/- 11.53, and the average BMI was 27.18 +/- 5.29. Some of the relevant lab values were noted, with the averages of the following given in parentheses: IgE (522.94 +/- 876.54), eosinophils (0.764 +/- 1.73), FENO (35.54 +/- 39.74).

**Table 1:** General characteristics of our patients. IgE: Immunoglobulin E; FeNO: Fractional Exhaled Nitric Oxide; FVC: Forced Vital Capacity; FEV1: Forced Expiratory Volume in One Second.

	Mean	SD	Median	Mode	Variance
Age	41.89	11.5	40	34	133.01
BMI	27.18	5.29	27	27	28.03
IgE	522.94	876.5	215.8	42.3	768335.2
Eosinophils	0.76	1.73	0.2	0.1	3.027
FENO	35.54	39.74	22	9	1579.3
FVC	93.5	23.37	93.5	95	546.5
FEV1	86.02	19.69	88	88	387.86

Analyzing qualitative variables across the three grades of MEF-25 severity

In the study, the MEF-25 values were graded based on their severity. The three grades given are moderate (51-80), severe (35-50), and very severe (1-34).

Tables 2-4 present a comparison of various variables relevant to patients with asthma, assessing significant differences across the three MEF-25 severity grades. Our statistical analysis revealed significant differences in the distribution of IgE, FENO, FVC, and FEV1 across these categories. Using the Independent Samples Kruskal-Wallis Test, we found that the distribution of IgE (p=0.18), FeNO (p=0.16), FVC (p=0.01), and FEV1 (p<0.0001) differed significantly among the MEF-25 categories.

**Table 2:** General characteristics of patients with moderate MEF-25 severity. IgE: Immunoglobulin E; FeNO: Fractional Exhaled Nitric Oxide; FVC: Forced Vital Capacity; FEV1: Forced Expiratory Volume in One Second; MEF-25: Maximal Expiratory Flow at 25%.

Moderate MEF-25 severity	Mean	Median	Mode	SD	Variance
BMI	26.81	26	26	5.12	26.21
IgE	421.73	156.9	42.3	761.59	580014.25
Eosinophils	0.72	0.2	0.1	1.69	2.86
FENO	27.89	17.5	9	29.93	896.53
FVC	96.24	95	90	18.26	333.45
FEV1	92.81	92	88	13.44	180.73

**Table 3:** General characteristics of patients with severe MEF-25 severity. IgE: Immunoglobulin E; FeNO: Fractional Exhaled Nitric Oxide; FVC: Forced Vital Capacity; FEV1: Forced Expiratory Volume in One Second; MEF-25: Maximal Expiratory Flow at 25%.

Severe MEF-25 severity	Mean	Median	Mode	SD	Variance
BMI	27.44	27	27	5.35	28.56
IgE	595.71	243.5	521.1	1135.03	1288206.9
Eosinophils	0.96	0.2	0.1	2.18	4.77
FENO	43.9	28.5	10	48.74	2375.45
FVC	93.92	94.5	84	17.19	295.42
FEV1	84.97	85.5	92	13.5	182.18

**Table 4:** General characteristics of patients with very severe MEF-25 severity. IgE: Immunoglobulin E; FeNO: Fractional Exhaled Nitric Oxide; FVC: Forced Vital Capacity; FEV1: Forced Expiratory Volume in One Second; MEF-25: Maximal Expiratory Flow at 25%.

Very Severe MEF-25 Severity	Mean	Median	Mode	SD	Variance
BMI	27.92	27	24	5.76	33.23
IgE	705.46	666.5	565.5	655.61	429824.43
eosinophils	0.61	0.35	0.1	1.08	1.17
FENO	42.68	28	9	43.72	1911.61
FVC	84.22	81	58	40.38	1630.58
FEV1	66.66	62.5	38	30.13	907.74

Tables 2-4 show that the mean values of IgE and FENO increase as MEF-25 values decrease, indicating an inverse relationship. Within the MEF-25 range of 35-80, FEV1 values remain within the normal range and only show a substantial drop in the very severe category.

In Tables 5-12, we assessed the variables of sex, smoking status, pet ownership, and common asthmatic symptoms, including cough, shortness of breath (SOB), chest tightness, and wheeze.

**Table 5:** Distribution of MEF-25 severity levels by sex (male and female). MEF-25: Maximal Expiratory Flow at 25%.

Sex	Male	Female
Moderate	66	53
Severe	30	38
Very Severe	10	28

**Table 6:** Distribution of MEF-25 severity levels by smoking status. MEF-25: Maximal Expiratory Flow at 25%.

Smoking status	Current	Ex-smoker	Never
Moderate	13	6	66
Severe	2	3	48
Very Severe	4	5	25

**Table 7:** Distribution of MEF-25 severity levels by pet ownership status (with or without pets). MEF-25: Maximal Expiratory Flow at 25%.

Pet Ownership Status	Yes	No
Moderate	17	56
Severe	13	33
Very Severe	12	18

**Table 8:** MEF-25 severity levels among patients with and without shortness of breath. MEF-25: Maximal Expiratory Flow at 25%.

Shortness of Breath	Present	Absent
Moderate	75	41
Severe	44	20
Very Severe	28	10

**Table 9:** MEF-25 severity levels among patients with and without cough. MEF-25: Maximal Expiratory Flow at 25%.

Cough	Present	Absent
Moderate	97	19
Severe	51	14
Very Severe	34	4

**Table 10:** MEF-25 severity levels among patients with and without chest tightness. MEF-25: Maximal Expiratory Flow at 25%.

Chest Tightness	Present	Absent
Moderate	48	68
Severe	32	33
Very Severe	20	18

**Table 11:** MEF-25 severity levels among patients with and without wheeze. MEF-25: Maximal Expiratory Flow at 25%.

Wheeze	Present	Absent
Moderate	49	67
Severe	40	25
Very Severe	26	12

**Table 12:** Values of significance for Pearson chi-square, Phi, and Cramer for each category. SOB: Shortness of breath.

Different Categories	Pearson Chi-square	Phi	Cramer's V
Sex	10.17 (p-0.006)	0.213 (p=0.0006)	0.213 (p=0.006)
Smoking status	8.467 (p-0.206)	0.219 (p=0.206)	0.155 (p=0.206)
Pet	2.934 (p=0.231)	0.14 (p-0.231)	0.14 (p=0.231)
SOB	4.247 (p=0.374)	0.137 (p=0.374)	0.097 (p=0.374)
Cough	3.979 (p=0.409)	0.133 (p=0.409)	0.094 (p=0.409)
Chest tightness	3.799 (p=0.434)	0.130 (p=0.434)	0.092 (p=0.434)
Wheeze	12.811 (p=0.012)	0.239 (p=0.12)	0.169 (p=0.12)

A significant difference in gender distribution was found across the three MEF-25 groups (p=0.006), with Phi and Cramer's V analysis indicating a p-value of 0.23.

Tables 5-12 also show our assessment of smoking status, pet ownership, and asthmatic symptoms. Chi-square analysis revealed no significant association between MEF-25 categories and the proportion of smokers (p=0.206) or patients with pets (p=0.23).

However, we found a statistically significant association between the symptom of wheeze and MEF-25 severity (p=0.012). There was no significant association between MEF-25 severity and the symptoms of SOB (p=0.43), cough (p=0.40), or chest tightness (p=0.37).

Symptom-wise response of SAD patients to beclomethasone-formoterol****

Another outcome of our study was to assess the response of patients to Beclomethasone-formoterol. In Table 13, we examine the follow-up data 1-6 weeks after patients began using the inhaler to determine whether their symptoms such as SOB, cough, chest tightness, and wheeze persisted. Note that patients can have one or more of these symptoms simultaneously.

**Table 13:** Frequency of the symptoms after using beclomethasone-formoterol for more than two weeks.

		Frequency	Percentage
Shortness of breath	Persistent	23	21.30%
	Non persistent	85	78.70%
Cough	Persistent	34	32.10%
	Non persistent	72	72%
Chest tightness	Persistent	11	10.50%
	Non persistent	94	89.50%
Wheeze	Persistent	11	10.60%
	Non persistent	93	89.40%

In our patient population, 1-6 weeks after starting Beclomethasone-formoterol therapy, 23 patients (21.3%) had persistent SOB, while 85 patients (78.7%) had nonpersistent SOB. For cough, 34 patients (32.1%) experienced persistent symptoms, and 72 patients (67.9%) had nonpersistent symptoms. For chest tightness, 11 patients (10.5%) reported persistent symptoms, and 94 patients (89.5%) had nonpersistent symptoms. Finally, for wheeze, 11 patients (10.6%) had persistent symptoms, while 93 patients (89.4%) had nonpersistent symptoms (Table 13).

## Discussion

Patients with SAD tend to have poorly controlled asthma and use higher mean doses of ICS compared to those without SAD [12]. This emphasizes the significance of our study, as SAD remains a relatively new and underexplored area of research.

General characteristics and risk factors of patients with SAD 

Our study initially examined the general characteristics of patients with small airway disease. We then explored further, examining the characteristics and risk factors of our patients based on their MEF-25 levels.

One study noted that patients with SAD were more likely to be female, older, overweight, non-atopic, current or former heavy smokers, to have longer asthma duration, and higher levels of serum eosinophils and FeNO [12].

Our results show a significant difference in gender across the three MEF-25 groups (p=0.006, CI: 95%) (Table 3); however, this difference is most likely due to a sampling artifact. Furthermore, Phi and Cramer’s V analysis showed a value of 0.23, indicating that this sampling artifact is weakly associated with the MEF-25 categories and can therefore be reasonably ignored.

We further evaluated these risk factors (Table 3) and found that they remained consistent across different severities of MEF-25. The average BMI of our patients was 27.18 ± 5.29, indicating that the majority were overweight. Additionally, the average FeNO levels were 35.54 ± 39.74, indicating elevated levels. These findings confirm and reaffirm previous studies regarding the risk factors of patients with small airway disease.

Role of MEF-25 in SAD 

Primary indicators for evaluating small airway function include FEF at 50%, 75%, and 25-75% (also known as maximum mid-expiratory flow, or MMEF), with FEF often used interchangeably with maximal expiratory flow (MEF). SAD is typically identified when two out of these three indicators fall below 65% of the predicted value [13]. However, it is important to note that MEF25-75% can be significantly influenced by the degree of expiratory effort, which can potentially be a limitation in its clinical application [14,15].

One of the aims of our research is to evaluate the role of MEF-25 in detecting early small airway obstruction. MEF-25 corresponds to the final 25% of exhalation, which originates from the most distal bronchioles. Its readings can decrease even when other spirometry measurements, such as FEV1 and FVC, fall within normal limits. As a result, MEF-25 is a highly valuable indicator for identifying SAD in individuals presenting with asthma-like symptoms.

A study found that assessing FEF25-75 percent predicted offers advantages over FEV1 percent predicted and FEV1/FVC percent predicted in diagnosing pediatric asthma. The results showed that FEF25-75 percent predicted correlates with bronchodilator responsiveness in asthmatic children with normal FEV1, indicating airway dysfunction despite normal FEV1. Thus, assessing FEF25-75 percent predicted in pediatric asthma clinical trials is crucial for detecting clinically significant, reversible airflow obstruction. Other studies also indicate that FEF25-75 is more sensitive than FEV1 in identifying symptomatic asthma in adults [16-18].

Role of MEF-25-75% and FENO in detecting airway hyperresponsiveness

Several methods exist for evaluating airway hyperresponsiveness (AHR), with the most practical being the assessment of MEF or FEF 25-75% and the FENO. AHR is characterized by the airways narrowing disproportionately in response to stimuli that minimally affect healthy individuals [19].

Our research demonstrated substantial variation in the levels of FENO across the three distinct categories of MEF-25, which indicates that as inflammation and constriction of the small airways increase, higher levels of exhaled nitric oxide (FENO) are produced, while lower levels of MEF-25 are observed in spirometry. A retrospective cross-sectional study by Chinese researchers [20] used FEF-50% or FEF 25-75% to diagnose SAD in symptomatic patients with normal FEV1. They found a significantly elevated probability of AHR in individuals with FEF 25-75% <84.4%, FEF 50% <76.8%, and FENO >41 ppb. These findings, in conjunction with our own results, highlight the crucial role and importance of MEF-25 and FENO in detecting AHR.

Significance of wheeze in SAD

We discovered a striking correlation between the symptom of wheezing and lower MEF-25 values (<80%). Pearson Chi-square analysis revealed that the likelihood of wheezing increases as MEF-25 values decrease. Our findings clearly indicate that reduced MEF-25 levels are associated with a higher incidence of wheezing. This is noteworthy, as wheezing is a sound uniquely generated by the smaller airways or bronchioles, suggesting that narrowing of these airways is associated with reduced MEF-25 levels. Wheeze can be detected during history-taking or physical examination, highlighting the crucial importance of thorough history-taking and clinical examination in evaluating the diagnosis of SAD or obstruction [21].

Another study found that a questionnaire and basic patient characteristics can predict SAD in asthma patients. Notably, even without respiratory test results, a specific questionnaire item ("I sometimes wheeze when I am sitting or lying quietly") and factors like age, age at asthma diagnosis, and BMI effectively identified patients more likely to have SAD [22]. This shows that there is a significant association between wheezing specifically and SAD. In contrast, other symptoms of SAD, such as cough, chest tightness, and shortness of breath, are not exclusive to the lower airways and may also arise from the involvement of the upper airways or other causes. Therefore, it can be said that the historical and physical examination finding of wheeze is strongly indicative of SAD and should prompt us to choose treatments that penetrate deeply into the lungs as proven by finer inhaler formulations delivered with a spacer.

Management of SAD with inhalers containing ICS and LABA 

Moving on to the management of SAD patients, international treatment guidelines recommend using an inhaler with both ICS and a LABA for relieving SAD symptoms. In our study, we employed a beclomethasone-formoterol combination inhaler with a spacer. The following literature review highlights key studies on the benefits of extra-fine ICS for asthmatic patients with SAD.

Numerous studies have shown that asthmatic patients with SAD benefit significantly from extra-fine ICS, such as beclomethasone-formoterol. These medications improve lung function, airway responsiveness, symptoms, exacerbation rates, and overall asthma control [23, 24]. The extra-fine formulation enhances the drug's impact by delivering it more deeply into the distal airways, increasing its effectiveness. Beclomethasone-formoterol has been observed to cause faster bronchodilation lasting up to an hour, likely due to the smaller particles allowing deeper lung penetration and quicker action. Additionally, studies have demonstrated that an extra-fine particle formulation of beclomethasone can be used at half the dose of the large-particle formulation while achieving equally good clinical outcomes. These extra-fine formulations effectively target the small airways and may offer additional clinical benefits compared to large-particle treatments [25-27].

The benefits of using a spacer have been well documented in current literature. Despite this, not a lot of clinical practice emphasizes its use in reality. The spacer allows for more streamlined and even delivery of medication and is capable of penetrating the deeper, more inaccessible distal airways. This helps to minimize the negative effects frequently linked to inhaler medication deposition in the mouth, tongue, and oropharynx and significantly lessens the financial burden from the resulting medication waste. Our data provides real-life evidence that endorses the use of a spacer in effectively relieving symptoms. This goes on to say that physicians should not underestimate the efficacy of a spacer and actively encourage and ensure the use of a spacer while prescribing inhalers.

In our study, we evaluated the response of patients to a beclomethasone-formoterol inhaler used with a spacer, assessing the persistence of their symptoms. One to six weeks after starting beclomethasone-formoterol therapy, 21.3% of patients had persistent shortness of breath, 32.1% had a persistent cough, 10.5% had persistent chest tightness, and 10.6% had persistent wheeze. The majority of patients reported significantly improved symptom control and non-persistence of their symptoms, reinforcing existing literature on the superior efficacy of beclomethasone-formoterol.

Therefore, it can be stated that the formulation, particle size, and deposition characteristics of ICS play crucial roles in the real-life effectiveness of asthma therapy, especially for those with small airway involvement. Our research, consistent with previous studies, showed that using an ultra-fine formulation of beclomethasone formoterol improved symptoms such as cough, wheeze, dyspnea, and chest tightness, which typically resolved within 1-6 weeks of treatment initiation. This targeted treatment approach is especially beneficial for individuals with clinically significant small airway inflammation, enhancing their likelihood of achieving better outcomes.

Limitations and future research

The limitations of our study lie in its retrospective and observational design, which may have influenced the accuracy of some results and made it impossible to compare and correlate our findings with current standards for measuring SAD, such as oscillometry.

We endorse MEF-25 as a useful tool to screen for SAD and hope future studies assess its sensitivity compared to MEF-50 and MEF-75. A point to note regarding parameters such as MEF-25 is that while they are easy to obtain, they rely heavily on FVC, which is subject to the patient’s expiratory effort and therefore poorly reproducible.

We reported symptomatic improvement as recorded in physicians' notes as the final outcome and acknowledge the need to reassess outcomes with standardized control assessment tools in future studies. Also, the adherence of patients to the prescribed inhaler with a spacer needs to be more concretely assessed in future research.

Furthermore, we report that in addition to beclomethasone-formoterol, some patients may have been prescribed additional medications such as cough syrups and mucolytic agents which could act as potential confounders.

## Conclusions

In conclusion, our study demonstrates that using MEF-25 as an early marker for small airway dysfunction in outpatient settings is practical, cost-effective, and valuable, especially in patients presenting with symptoms like cough, shortness of breath, wheeze, or chest tightness that may not be detected through standard spirometry. We found a strong correlation between lower MEF-25 values and increased FENO levels, highlighting the role of MEF-25 in identifying more severe airway impairment.

Moreover, our findings provide real-life evidence of significant symptomatic improvement with the use of ultra-fine beclomethasone-formoterol via a spacer, underscoring its efficacy in targeting small airways. This approach not only enhances patient outcomes but also reduces the broader economic and clinical burden of progressive airway disease.
